# Temporal and Spatial Profiling of *Escherichia coli* O157:H7 Surface Proteome: Insights into Intestinal Colonisation Dynamics In Vivo

**DOI:** 10.3390/proteomes13040052

**Published:** 2025-10-10

**Authors:** Ricardo Monteiro, Ingrid Chafsey, Charlotte Cordonnier, Valentin Ageorges, Didier Viala, Michel Hébraud, Valérie Livrelli, Alfredo Pezzicoli, Mariagrazia Pizza, Mickaël Desvaux

**Affiliations:** 1 Instituto de Investigação e Inovação em Saúde-i3S, Universidade do Porto, 4150-564 Porto, Portugal; 2 INRAE, Université Clermont Auvergne, UMR0454 MEDIS, 63000 Clermont-Ferrand, France; 3 GSK, 53100 Siena, Italy; 4 M2iSH, UMR 1071 INSERM, University of Clermont Auvergne, 63001 Clermont-Ferrand, France; 5 INRAE, Plateforme d’Exploration du Métabolisme—Composante Protéomique (PFEMcp), 63122 Saint-Genès-Champanelle, France; 6 Centre for Bacterial Resistance Biology, Department of Life Sciences, Imperial College, London SW7 2AZ, UK

**Keywords:** EHEC O157:H7, surface proteome, host colonization

## Abstract

Background: EHEC O157:H7 causes severe gastrointestinal illness by first colonizing the large intestine. It intimately attaches to the epithelial lining, orchestrating distinctive “attaching and effacing” lesions that disrupt the host’s cellular landscape. While much is known about the well-established virulence factors, there are much to learn about the surface proteins’ roles in a living host. Methods: This study presents the first in vivo characterisation of the surface proteome, i.e., proteosurfaceome, of *Escherichia coli* O157:H7 EDL933 during intestinal infection, revealing spatial and temporal adaptations critical for colonisation and survival. Using a murine ileal loop model, surface proteomic profiles were analysed at early (3 h) and late (10 h) infection stages across the ileum and colon. Results: In total, 272 proteins were identified, with only 13 shared across all conditions, reflecting substantial niche-specific adaptations. Gene ontology enrichment analyses highlighted dominant roles in metabolic, cellular, and binding functions, while subcellular localisation prediction uncovered cytoplasmic moonlighting proteins with surface activity. Comparative analyses revealed dynamic changes in protein abundance. Conclusions: These findings indicate a coordinated shift from stress adaptation and virulence to nutrient acquisition and persistence and provide a comprehensive view of EHEC O157:H7 surface proteome dynamics during infection, highlighting key adaptive proteins that may serve as targets for future therapeutic and vaccine strategies.

## 1. Introduction

Enterohaemorrhagic *Escherichia coli* (EHEC) O157:H7 is a significant foodborne pathogen responsible for severe gastrointestinal diseases in humans [1]. It has been recognised as a leading cause of outbreaks associated with the consumption of contaminated food, particularly, undercooked beef and raw vegetables [1,2,3]. The clinical manifestations of this pathogen range from mild diarrhoea to haemorrhagic colitis. In severe cases, it can cause life-threatening haemolytic uremic syndrome (HUS), which is characterised by haemolysis, acute kidney failure, and thrombocytopenia. The ability of EHEC O157:H7 to cause such severe outcomes is linked to its virulence factors, particularly the production of shiga toxins, and the ability to intimately adhere to host intestinal epithelial cells [1,3,4].

EHEC O157:H7 primarily colonises the large intestine, where it attaches to the epithelial lining, causing distinctive “attaching and effacing” (A/E) lesions. These lesions result in the destruction of microvilli and rearrangement of the actin cytoskeleton, disrupting normal cellular function and leading to characteristic symptoms of infection [4]. Understanding how EHEC interacts with host tissues at the molecular level is essential to develop therapeutic strategies to mitigate or prevent these infections.

Several studies have been devoted to understanding the molecular basis of the pathogenicity of EHEC O157:H7. Shiga toxins (Stx1 and Stx2) are well-established primary virulence factors responsible for systemic complications associated with EHEC infections, including HUS. These toxins inhibit protein synthesis in host cells by targeting ribosomal RNA, leading to cell death, particularly in the kidney [3,4,5,6]. The locus of the enterocyte effacement (LEE) pathogenicity island encodes a type III secretion system (T3SS), which is critical for the ability of the bacterium to inject effector proteins into host cells [4,7]. These effectors manipulate the host cell cytoskeleton and signalling pathways, facilitating the formation of A/E lesions [3,5].

Despite the wealth of knowledge on these key virulence factors in vitro, little is known about the role of other bacterial surface proteins in EHEC pathogenesis in vivo. Surface proteins play a critical role in mediating the initial interaction between EHEC and host intestinal epithelial cells, facilitating adhesion, colonisation, immune evasion, and persistence within the host [1,2,3,4,5,6,7,8,9,10,11,12,13]. Several bacterial surface proteins have been identified in EHEC O157:H7 that contribute to its virulence, including intimin, a critical adhesin that binds to the host cell receptor Tir (translocated intimin receptor) [12,14,15]. Tir is delivered into host cells via the T3SS, where it becomes embedded in the host cell membrane, allowing intimin to establish intimate attachment [16]. This interaction is essential for the formation of A/E lesions and the ability of bacteria to colonise the gut [5]. Other notable surface proteins include flagella, which not only contribute to motility but also play a role in early adhesion to host tissues [17]. Additionally, outer membrane proteins (OMPs), such as OmpA and OmpC, are involved in maintaining the structural integrity of the bacterial membrane and have been shown to interact with host immune components, aiding immune evasion [4,8,12,13,18,19,20,21,22,23,24]. Furthermore, studies have indicated that EHEC can form biofilms on surfaces, including the intestinal epithelium, which enhances bacterial persistence and resistance to host immune responses and environmental stressors [4,7,12]. Curli fibers, which are extracellular amyloid fibers produced by EHEC, are also implicated in biofilm formation and have been shown to bind to host cells, further promoting colonisation [5,8,25].

Although these findings provide important insights into the surface proteins of EHEC O157:H7, a comprehensive understanding of the full range of surface proteins and their dynamic alterations during infection remains limited. Surface proteomics offers a powerful approach for characterising the complete array of proteins present on the bacterial surface, allowing for the identification of previously unrecognised proteins that may contribute to virulence, host interaction, and immune evasion [11,26,27]. Moreover, surface proteins are often the first bacterial components encountered by the host immune system, making them promising targets for vaccine development and therapeutic intervention [10,22,28].

In this study, we investigated the proteosurfaceome of EHEC O157:H7, i.e., the bacterial surface proteins expressed during infection of the intestine, considering both the ileum and colon, in a mouse model. By comparing the proteosurfaceome of EHEC O157:H7 at early and late infection stages in different segment of the intestine, we provide the first identification of surface proteins expressed in vivo that potentially play critical roles in host colonisation, immune evasion, and bacterial persistence.

## 2. Materials and Methods

### 2.1. Bacterial Growth Conditions

*E. coli* O157:H7 strain EDL933 was used in this study [29]. Pre-cultures were grown overnight in BHI liquid medium at 37 °C with orbital shaking in triplicate. After 1:100 (*v*/*v*) dilution, the bacterial cultures were grown under the same conditions until the late exponential phase (OD_600_ = 0.8 nm). Bacterial cells were harvested by centrifugation (4000× *g*, 5 min, 4 °C) and washed with PBS.

### 2.2. Mice Ileal Loop Assay

In vivo adaptations of the surface proteome of EHEC bacteria were studied using mouse ileal loops as previously described [9]. Briefly, mice (n = 6, 3 for ileum and 3 for colon) were starved 24 h before the operation, anesthetised, and the abdominal cavity was exteriorised through a midline incision. A 6 cm ileal segment in the case of the ileum and the entire colon was isolated and ligated. These sections were then inoculated with 5 × 10^8^ CFU/mL of *E. coli* O157:H7 strain EDL933. Three hours (early infection) and 10 h (late infection) after injection, the mice were euthanised by cervical dislocation according to the animal care procedure. The intestinal and colonic mucosa were recovered, washed, and surface-scraped. Both washed and scrapped materials were pooled and stored in PBS (10 mM, pH 8) for further protein biotinylation.

### 2.3. Ethics Statement

Animal studies were performed in compliance with French and European regulations on the care and protection of laboratory animals (EC Directive 2010/63, French Law 2013–118, 6 February 2013). All experiments were approved by the Comité d’Ethique en Matière d’Expérimentation Animale Auvergne (CEMEAA) and registered under reference C2EA-02.

### 2.4. Animals

Adult (5–6 weeks old) male FVB mice were purchased from Charles River Laboratories. Animals were housed in the Université d’Auvergne Medical School animal facility accredited by the French Ministry of Agriculture for performing experiments on live rodents (Agreement 2014 C63 113 15).

### 2.5. Biotinylation of Bacterial Cell Surface Proteins and Protein Affinity Purification

Labelling and isolation of bacterial cell-surface proteins were performed as previously described using Sulfo-NHS-SS-biotin (Sulfo-succinimidyl biotin-amidoethyl dithio-propionate) [26]. Biological material, including bacterial cells from the infected animals, was maintained in PBS (10 mM, pH 8) and incubated with Sulfo-NHS-SS-biotin (1% *w*/*w*) for 5 min at room temperature. The excess biotinylation reagent was quenched by three washes with a solution of 10 mM PBS, pH 8 and 500 mM glycine (4000 g, 5 min, 4 °C), and cells were resuspended in lysis buffer (10 mM PBS pH 8, 1 mM PMSF, 1% *v*/*v* Triton^TM^ X-100, Merck KGaA, Darmstadt, Germany). For reaction control, cells were incubated with 10 mM PBS (pH 8) instead of biotin reagent and underwent the same procedure. Cell disruption was performed using Fast-prep (MP Biomedicals) with two steps of 20 s at 6 m/s, and cell debris from cell lysis was discarded by centrifugation (20,000× *g*, 30 min, 4 °C). Labelled proteins in the supernatant were purified by affinity purification over a column containing NeutrAvidin agarose resin (Pierce Thermo Scientific, Waltham, MA, USA.) following the manufacturer’s instructions with some modifications in the washing phase. Briefly, the columns were equilibrated with wash buffer (10 mM PBS, pH 8, 1% NP-40), and the same volume of each protein sample as well as the control was loaded and kept in contact with the resin at room temperature for 15 min. Unlabelled proteins were washed away using 10 column volumes of wash buffer. Finally, labelled proteins were eluted (2% SDS, 20% glycerol, 62.5 mM Tris-HCl, 50 mM DTT, 5% β-mercaptoethanol). Sulfo-NHS-SS-biotin is a membrane-impermeable reagent that covalently couples to primary amines, primarily lysine side chains and N-termini, on proteins accessible at the bacterial surface. This approach enables the selective enrichment of surface-exposed proteins for downstream affinity purification. However, proteins with a higher density of accessible lysines may be preferentially labeled and thus overrepresented, while proteins with fewer exposed amines or those shielded within complexes may be underrepresented [30].

### 2.6. Mass Spectrometry Analysis

All eluted samples were loaded onto an SDS-PAGE gel and concentrated into a single band. The excised bands were then washed in 25 mM ammonium bicarbonate with 5% acetonitrile for 30 min and twice in 25 mM ammonium bicarbonate with 50% acetonitrile for 30 min. Reduction and alkylation reactions were performed with 10 mM DTT and 55 mM iodoacetamide solutions, respectively, before the bands were dehydrated with 100% acetonitrile. The samples were hydrolysed overnight with 10 ng/µL trypsin (Promega, Madison, WI, USA), and the peptides were extracted with 100% acetonitrile before being concentrated (SpeedVac, Thermo Fisher Scientific, Waltham, MA, USA) and resuspended in the same volume.

Hydrolysed samples were analysed by nanoLC-MS/MS using an Ultimate 3000 system (Dionex Thermo Fisher, Waltham, MA, USA) coupled with an ION TRAP mass spectrometer (LTQ Velos, Thermo Scientific, Waltham, MA, USA). After desalting on a C18 pre-column (300 μm, 5 mm), peptides were separated on an analytical C18 nanocolumn (75 μm, 15 cm) using a 50 min gradient from 10% to 40% solvent A (80/20 acetonitrile/H_2_O, 0.5% formic acid) in solvent A (100% H_2_O, 0.1% formic acid). The eluate was electrosprayed onto the mass spectrometer. MS data were acquired in data-dependent acquisition mode (a top 10 method). Full MS scans were acquired in the *m*/*z* range 300–2000. The spray voltage was set to 1.9 kV, with a capillary temperature of 275 °C. The most intense precursor ions were selected for collision-induced dissociation (CID) with a normalized collision energy of 35% and an isolation window of 2 *m*/*z*. Dynamic exclusion was enabled with a repeat count of 1 and an exclusion duration of 30 s.

Each experiment was performed in triplicate (biological replicates), with two runs per sample (technical repeats).

### 2.7. Protein Identification and Comparison

Protein identification was performed using the *E. coli* O157:H7 strain EDL933 database (KEGG Genome T00044, 5449 protein entries) and the Mascot search engine. Only canonical proteins or ORF products rather than proteoforms were identified. Peptides were validated for a Mascot score permitting to obtain a false discovery rate (FDR) at below 5% and proteins were identified when at least two peptides matched significantly (score higher than 20) in the database with search parameters set to 10 ppm for peptide and 0.5 Da for fragment mass tolerance. Single peptides were subjected to MS/MS fragmentation for validation.

The minimal Mascot score threshold was adjusted to 25. Any individual peptide score above this threshold indicates a significant match for the reported sequence at *p* < 0.05.

Only canonical proteins or ORF products rather than proteoforms were identified respective to the nature and complexity of the samples.

Comparison of protein abundance was performed following the standard workflow provided by Progenesis QI for proteomics applying the label-free relative quantification method using Hi-N mode (Nonlinear Dynamics, Waters); basically, the MS1 survey scans are used for ion abundance quantification where peptide ions falling within the robust estimation limits are used to calculate the normalisation factor and selected as normalisation reference to compare peptidic signals between runs. For each subprotoeme, differential protein quantification was considered significant for fold abundance greater than 1.5 and *p*-value lower than 0.05. FDR (false discovery rate) approach was applied to overcome the issue of multiple hypothesis testing and q-values (adjusted *p*-values) were further calculated.

### 2.8. Secretome Prediction and Gene Ontology Enrichment

Secretomic analysis was performed for LPS-diderm bacteria [31] using different software tools to predict protein transport pathways, subcellular localisations and protein categories. In silico predictions of protein subcellular localisation (SCL) were accessed using PSORTb v.3.0.2 [32] combined with SignalP v5.0 to identify export signal peptides [33] and SecretomeP v2.0 for prediction of non-classically secreted proteins [34] as well as KEGG (Kyoto encyclopedia of genes and genomes) [35] and literature survey for protein secretion pathways [10,36]. Identified proteins predicted as located at the cytoplasm were filtered for moonlight activity using MoonProt v2.0 [37]. Combining these different predictions with literature survey and Uniprot information, protein transport route was predicted for each protein (Table S1). The information of gene ontology (GO) for molecular function and biological process was retrieved from UniProtKB and QuickGO. Enrichment of overrepresented GO terms was performed using the Panther Classification System (v.17.0) up to the ancestor level 2 [38]. The Panther overrepresentation test (Fisher’s Exact test with FDR correction) was applied to assess statistical significance. For clarity of representation, the figures display the percentage of proteins assigned to each GO term.

## 3. Results

### 3.1. Intestinal Profiling of E. coli O157:H7 Surface Proteins

**Figure 1:**
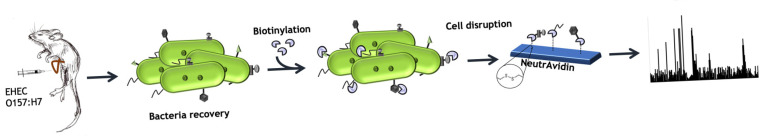
Workflow of the experimental approach used in this study. Mouse ileal loops and colon segments were infected with EHEC O157:H7 for early (3 h) and late (10 h) time points. Bacterial surface proteins were isolated by biotinylation and affinity purification, digested and analyzed by nanoLC-MS/MS, followed by protein identification/quantification (Mascot v2.6.0, Progenesis QI v3.0).

**Figure 2:**
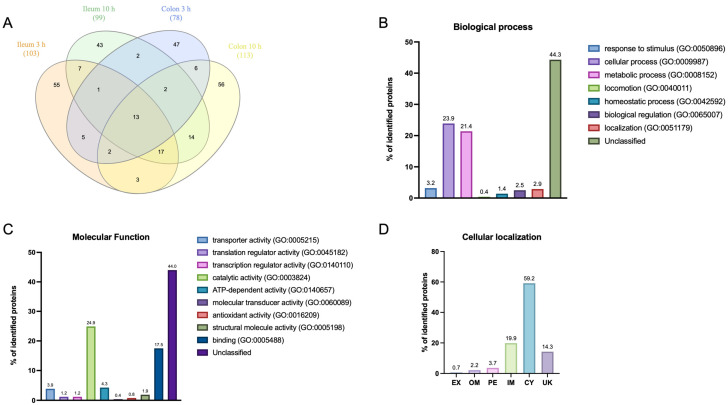
Distribution of identified surface proteins of *E. coli* O157:H7 EDL933 in the different experimental conditions. (**A**) Venn diagram comparing the number of identified proteins for the four experimental conditions and its overlay. (**B**) Percentage of unique proteins identified in the four experimental conditions and respective GO terms for biological process. Identified biological process terms included (i) GO:0050896 related to any process that results in a change in state or activity of a cell or an organism (in terms of movement, secretion, enzyme production, gene expression, etc.) as a result of a stimulus, (ii) GO:0009987 related to any process that is carried out at the cellular level, but not necessarily restricted to a single cell, (iii) GO:0008152 related to the chemical reactions and pathways, including anabolism and catabolism, by which living organisms transform chemical substances, (iv) GO:0040011 related to self-propelled movement of a cell or organism from one location to another, (v) GO:0042592 related to any biological process involved in the maintenance of an internal steady state, (vi) GO:0065007 related to any process that modulates a measurable attribute of any biological process, quality or function, and (vii) GO:0051179 related to any process in which a cell, a substance, or a cellular entity, such as a protein complex or organelle, is transported, tethered to or otherwise maintained in a specific location. (**C**) Percentage of unique proteins identified in the four experimental conditions and respective GO terms for molecular function. Identified molecular function terms included (i) GO:0005215 related to the directed movement of substances (such as macromolecules, small molecules, ions) into, out of or within a cell, across or in between cells, (ii) GO:0045182 related to any molecular function involved in the initiation, activation, perpetuation, repression or termination of polypeptide synthesis at the ribosome, (iii) GO:0140110 related to the function that controls the rate, timing and/or magnitude of gene transcription, (iv) GO:0003824 related to catalysis of a biochemical reaction at physiological temperatures, (v) GO:0140657 related to coupling of ATP hydrolysis to other steps of a reaction mechanism to make the reaction energetically favourable, (vi) GO:0060089 related to a molecular function in which an effector function is controlled by one or more regulatory components, (vii) GO:0016209 related to the inhibition of the reactions brought about by dioxygen (O_2_) or peroxides, (viii) GO:0005198 related to The action of a molecule that contributes to the structural integrity of a complex and (ix) GO:0005488 related to the selective, non-covalent, often stoichiometric, interaction of a molecule with one or more specific sites on another molecule. Enrichment analysis was performed with Panther to the ancestor level 2 of GO [38]. (**D**) Percentage of unique proteins identified in the four experimental conditions respective to predicted SCL according the gene ontology (GO) terms for cellular component, i.e., are either located at the cell envelope (GO: 0030313), namely at the IM (GO: 0005886), in the periplasm (PE; GO: 0042597), at the OM (GO: 0019867), or in the extracellular milieu (EX; GO: 0005576); it further includes proteins predicted as cytoplasmic (CY; GO: 0005737).

The present study aims to study the surface proteomic profile adaptations of EHEC O157:H7 in vivo. To provide an overview of the experimental design and analysis pipeline, we included a schematic representation of the workflow (Figure 1). This summarizes the sequence of steps from in vivo infection and sample recovery, through surface protein biotinylation and affinity purification, to mass spectrometry acquisition and protein identification/quantification. A comprehensive proteomic analysis was conducted to investigate the protein abundance profiles of *E. coli* O157:H7 in the intestine using mouse model. Four different experimental conditions were established to access the spatial-temporal adaptations: two sections of the intestinal track were isolated, an ileal segment (around 6 cm) and the colon; and two time points, namely at 3 h (early stage of infection) and at 10 h (late stage of infection). In total, 272 different proteins were identified among all the conditions. To highlight both common and specific protein adaptation during infection, a Venn diagram was built (Figure 2A) and shows the intersection of proteins identified in the four conditions studied. Only 13 proteins were common to all conditions, representing the core surface proteins. Each condition also has unique proteins indicating some spatial and temporal-specific adaptations, with 55 exclusives to the ileum at 3 h, 43 to the ileum at 10 h, 47 to the colon 3 h and 56 unique to the colon at 10 h post-infection.

A gene ontology (GO) enrichment analysis was conducted to categorise the unique proteins identified across experimental conditions. The analysis yielded insights into both biological processes and molecular functions (Figure 2B,C), illustrating the diversity of protein roles involved in EHEC O157:H7 infection of mice. Although the individual protein identities varied between intestinal sites and time points, the broad functional categories (e.g., cellular and metabolic processes, catalytic activity, binding) were consistently represented across all conditions. It should be noted that while the graphical representation focuses on the proportion of proteins per GO term for clarity, the underlying enrichment analysis was performed with Panther using Fisher’s Exact test with FDR correction.

Regarding the classified biological process, the majority of proteins were involved in cellular processes (GO:0009987), comprising 23.9% of the dataset, and metabolic processes (GO:0008152), accounting for 21.4%. This distribution suggests an active involvement of metabolic and cellular pathways during infection, potentially driving the physiological adaptations required for bacterial survival and interaction with host cells. Additional biological processes, although less represented, included response to stimulus (GO:0050896) at 3.2% and localisation (GO:0051179) at 2.9%, reflecting the bacteria ability to respond dynamically to environmental and cellular cues within the host environment.

In terms of molecular functions, catalytic activity (GO:0003824) was the most enriched, representing 24.9% of the proteins. This highlights the significant role of enzymes in supporting metabolic processes essential for bacterial adaptation and survival. Binding activity (GO:0005488) was also prominent at 17.5%, suggesting interactions with host proteins or molecules. Other notable functions included transporter activity (GO:0005215) (3.9%) and ATP-dependent activity (GO:0140657) (4.3%), indicative of proteins that may facilitate nutrient uptake and energy-dependent cellular activities. Small proportions were observed for antioxidant activity (GO:0016209) (1.9%) and regulatory activities related to transcription and translation (1.2% each), suggesting an adaptive response to oxidative stress and regulation at the gene expression level in response to the host environment.

Unique proteins were also predicted for their subcellular localisation (SCL). The largest proportion of proteins was found as cytoplasmic (CY), making up 59.2% of the total, indicating a high level of metabolic and regulatory activity likely occurring within this cellular compartment (Figure 2D). Interestingly around 9% (14 out of 161) of the CY proteins were also predicted having moonlighting activity at the surface of the cell (MoonProt 3.0 database). Protein predicted to be inner membrane protein (IM) also shows a significant proportion at 19.9%, suggesting that many proteins are involved in transport and signalling processes across the bacterial membrane. Other localisations include periplasmic (PE) with 3.7% of identified proteins, outer membrane (OM) at 2.2%, and extracellular (EX) at 0.7%, each representing a smaller fraction of the total. These smaller groups suggest roles in interactions with the environment, structural support, and host interaction. Proteins with unknown localisation (UK) constituted 14.3%, highlighting a subset of proteins whose exact cellular roles remain to be determined, which may warrant further investigation.

Also, among the identified proteins, signal peptide predictions revealed that 16% are likely secreted via classical or alternative secretion pathways. Specifically, 14% of the proteins were predicted to be translocated through the Sec pathway, including both standard signal peptides and lipoprotein-associated variants, while only 2% were predicted to be exported through the Tat pathway. These findings support the notion that a subset of the surface proteome is actively processed for extracellular localization, potentially playing crucial roles in host–pathogen interactions and environmental sensing.

### 3.2. Expression of Specific Surface Proteins of E. coli O157:H7 EDL933 Is Modified Depending on the Environment and the Stage of Infection

To investigate the temporal and special adaptations of the proteosurfaceome of *E. coli* O157:H7 EDL933 during mice infection, the abundances of identified proteins were compared across the four experimental conditions. The goal of this analysis was to understand both the common and unique protein abundance patterns associated with early and late infection stages within distinct intestinal sections. Comparative analyses, including differential abundance and clustering, were conducted to identify proteins that exhibit condition-specific abundance. Additionally, gene ontology (GO) enrichment and cellular localisation insights were used to infer the potential functional roles of these proteins in bacterial adaptation and survival during infection.

**Figure 3:**
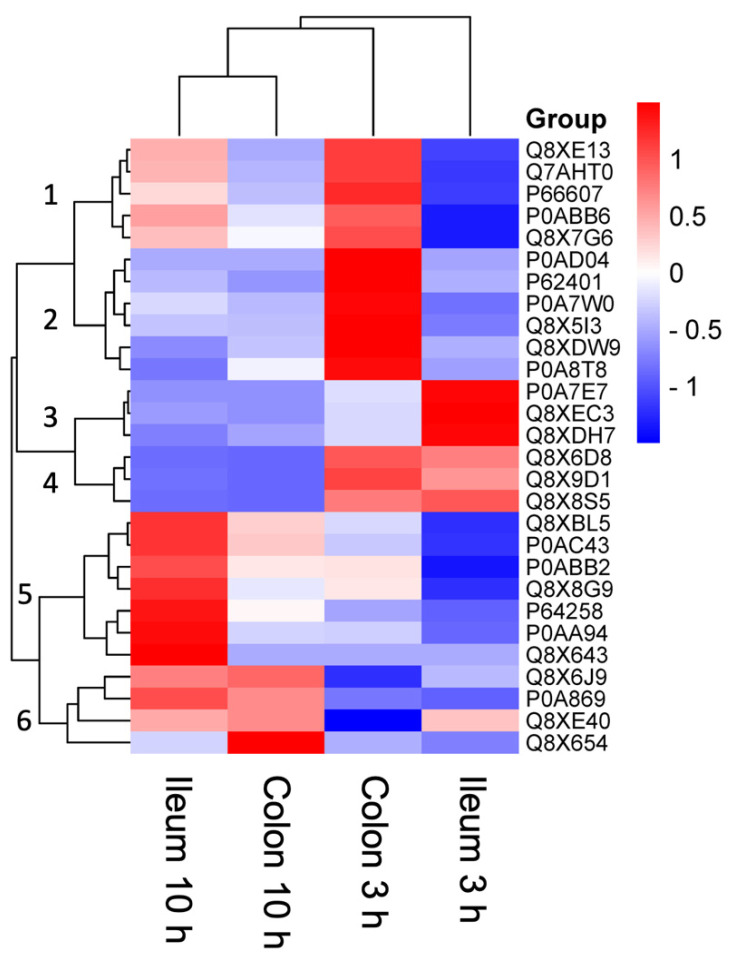
Heat map for differential proteins of *E. coli* O157:H7 EDL933 among the four experimental conditions: ileum 3 h, ileum 10 h, colon 3 h, and colon 10 h. Protein abundances were normalized across runs using the Progenesis Hi-N algorithm, log2-transformed, and scaled by Z-score to highlight relative differences across conditions. Hierarchical clustering of both proteins and conditions was performed using the Euclidean distance metric and average linkage. Only proteins with valid quantitative values in at least two biological replicates were retained; no imputation was applied. Statistical significance of differential abundance was determined by ANOVA with FDR correction, considering proteins with fold change >1.5 and adjusted *p* < 0.05 as significant. Shades from blue to red indicate relative normalized abundance (low to high). Dark red indicates higher protein abundance in a given condition compared to the others, and vice versa for dark blue. Dendrograms on the top and left sides represent hierarchical clustering of conditions and proteins, respectively, grouping them based on similarity in abundance patterns. Numbers from 1 to 6 identify the clusters of proteins differential abundant.

Regarding their relative differential abundances of identified proteins in *E. coli* O157:H7 EDL933 across four experimental conditions, 28 proteins were compared and divided into six clusters (Figure 3), i.e., (i) cluster 1 representing the specific proteins over-abundant in the colon at 3 h comparing to the ileum at 3 h post infection, (ii) cluster 2 representing the specific proteins over-abundant in the colon at 3 h post infection, (iii) cluster 3 shows the proteins over abundant in the ileum at 3 h post infection, (iv) cluster 4 the ones specific at early infection, (v) cluster 5 the proteins differentially abundant in early infection in comparison with late infection stage the ileum, and finally (vi) cluster 6 with the specific differentially abundant proteins between early and late infection in both the ileum and colon. This clustering of differential abundance in specific proteins demonstrates the proteins adaptation between the different stages of infection and different sections of the intestinal track.

Regarding the function associated with each one of the 28 proteins, a dynamic and region-specific variation in protein abundance could be observed highlighting distinct bacterial adaptation processes over time (Table 1).

At 3 h post-infection, bacterial protein abundance patterns in the colon reflected a clear prioritisation of anaerobic metabolism, stress resistance, and active protein synthesis, emphasising the physiological adaptations required for bacterial persistence in this environment. Proteins such as GlpB (Q8XE13) and FixC (Q7AHT0) were among the most highly abundant in the colon compared to the ileum. GlpB plays a key role in converting glycerol-3-phosphate to dihydroxyacetone phosphate using fumarate or nitrate as electron acceptors, highlighting a reliance on anaerobic respiratory pathways to generate energy. Similarly, FixC, potentially part of an electron transfer system, likely facilitates anaerobic carnitine reduction, suggesting that carnitine may be utilised as an alternative electron acceptor to support bacterial metabolism under oxygen-limiting conditions.

In addition to energy metabolism, a higher abundance of proteins involved in maintaining membrane integrity, amino acid metabolism, and stress resistance was observed in the colon at 3 h post-infection. PuuC (Q8X7G6), which catalyses the conversion of putrescine to glutamate via 4-aminobutanal, underscores the bacterial adaptation to nitrogen availability in the colon. The presence of YebS (P0AD04) suggests active transport mechanisms contributing to membrane stability, while CydC (Q8X5I3) connects sulfur metabolism to oxidative stress resistance and antibiotic tolerance. Additionally, AtpD (P0ABB6), the b-subunit of the ATP synthase, was highly abundant, underscoring the need for ATP production under anaerobic and nutrient-limited conditions. Indicative of active protein synthesis, ribosomal proteins such as RpsG (P66607) and RpsD (P0A7W0) were highly expressed, contributing to the assembly of the 30S ribosomal subunit and the regulation of rRNA transcription, together with RplE (P62401), a component of the 50S ribosomal subunit, facilitates the binding of 5S RNA and tRNA positioning during protein translation. Together, these findings suggest that bacterial growth in the colon during early infection relies on efficient ribosomal activity to maintain protein synthesis under challenging conditions.

In contrast, the bacterial proteomic profile in the ileum at 3 h post-infection was characterised by metabolic diversification, virulence activation, and osmotic stress adaptation. Proteins such as PyrG (P0A7E7), which catalyses the ATP-dependent amination of UTP to CTP, were highly abundant, reflecting a focus on nucleotide biosynthesis to support bacterial replication. UbiC (Q8XEC3), involved in the ubiquinone biosynthesis pathway, highlights bacterial reliance on ubiquinone for electron transport and energy production in the intestinal environment. The upregulation of TreA (Q8XDH7), an enzyme that facilitates trehalose breakdown under osmotic stress, suggests bacterial adaptation to fluctuating osmolarity within the intestinal lumen. A particularly notable finding was the elevated abundance of EivE (Q8X6D8), a key component of the Type III secretion system (T3SS) encoded within the SPI-1 locus. EivE regulates the secretion of effector proteins essential for host cell invasion, indicating early activation of virulence pathways in the ileum. Similarly, structural proteins like MreC (Q8X9D1), involved in maintaining rod-shaped bacterial morphology, and Csm (Q8X8S5), a methyltransferase, were highly expressed, further supporting bacterial adaptation to the intestinal environment.

By 10 h post-infection, bacterial proteomic dynamics shifted significantly, particularly in the ileum, where proteins involved in aerobic metabolism, DNA replication, and regulatory pathways were highly abundant. The increased expression of SdhA (P0AC43), a component of succinate dehydrogenase, indicates a metabolic shift towards aerobic respiration as the infection progressed. This was further supported by the upregulation of AtpA (P0ABB2), the a-subunit of ATP synthase, which reinforces the role of oxidative phosphorylation in energy production at this stage.

Proteins involved in DNA repair and replication, such as LigA (Q8XBL5), an NAD-dependent DNA ligase, were also enriched, reflecting bacterial responses to replication stress and DNA damage during late infection. Regulatory proteins including RcsD (Q8XE40), a key component of the Rcs signaling system, and in particular a LysR homolog (Q8X654), which exhibited an exceptionally high increase in abundance (>100-fold) during late infection, highlight a profound regulatory modulation. This dramatic upregulation strongly suggests that LysR-mediated transcriptional control plays a pivotal role in orchestrating bacterial adaptation to the host environment at this stage. Additionally, proteins like MraY (P64258), which catalyses the initiation of peptidoglycan biosynthesis, and YihI (Q8X8G9), a GTPase-activating protein likely involved in ribosome biogenesis, suggest active cell wall synthesis and ribosomal regulation during late infection.

Interestingly, in the colon at 10 h post-infection, fewer proteins exhibited significant enrichment compared to the ileum. Proteins such as TalA (P0A869), a transaldolase involved in the pentose-phosphate pathway, remained abundant, indicating continued metabolic maintenance despite reduced activity. Regulatory proteins, like RcsD (Q8XE40), persisted, albeit at lower levels, suggesting that bacterial responses in the colon at this stage were more subdued.

A significant trend was the reduced abundance of proteins such as EivE (Q8X6D8), MreC (Q8X9D1), and Csm (Q8X8S5) in the colon compared to the ileum, particularly during late infection stage. This suggests that virulence pathways and cell shape maintenance mechanisms, which were highly active in the ileum during early infection, became suppressed in the colon over time.

At 3 h post-infection, bacterial activity in the colon prioritised anaerobic metabolism, stress response, and ribosomal function, while the ileum displayed early activation of virulence factors and metabolic diversification. By 10 h, the proteomic profile exhibited a shift towards aerobic respiration, DNA repair, and transcriptional regulation, whereas bacterial activity in the colon appeared to decline, with only a subset of metabolic maintenance proteins remaining active.

## 4. Discussion

This study aimed at investigating the proteosurfaceome of EHEC O157:H7 EDL933 in response to the temporal and spatial adaptations of this pathogen in the intestine. We have previously described the modifications of EHEC O157:H7 subproteomes in different in vitro conditions, proving this pathogen can change its arsenal of virulence and fitness factors in order to better adapt and survive in different growth conditions [11]. However, no evidence of proteomic adaptation in vivo in response to host-specific environmental conditions was available to date. By comparing bacterial protein abundance between early (at 3 h post-infection) and late (at 10 h post-infection) stages of infection within two distinct intestinal regions, i.e., the ileum and colon, we sought to characterise key metabolic, stress response, and virulence-associated proteins that enable EHEC to colonise, adapt, and persist within the gastrointestinal tract. The proteosurfaceome was specifically targeted due to its critical role in bacterial–host interactions, including nutrient acquisition, immune evasion, and adhesion to host tissues [39,40].

However, performing proteomic studies in vivo using infection models is especially challenging, primarily due to the overwhelming abundance of host-derived proteins compared to bacterial proteins. This disparity often masks pathogen-specific signals, making detection and quantification difficult, especially for low-abundance proteins. The dynamic nature of bacterial populations within host tissues further complicates the studies, as pathogen heterogeneity and tissue localisation can influence proteomic profiles [41,42]. For these reasons, only a handful of studies have been conducted in vivo to date [42,43,44]. While previous proteomic studies on enteric pathogens such as *Salmonella* and *Staphylococcus aureus* have demonstrated the potential of this approach to reveal pathogen adaptations during host infection [39,45], these investigations were confined to specific tissues or biopsies in disease models such abscess formation. In contrast, our work applies this methodology to EHEC within a physiologically relevant in vivo model, capturing a pathogen with unique virulence mechanisms, host tropism, and niche-specific responses. By doing so, we extend the application of in vivo proteomics beyond what has previously been explored, offering novel insights into EHEC-specific host–pathogen dynamics and infection-stage-dependent adaptation that had not yet been characterized at the proteome level.

In our study, surface proteins were enriched by Sulfo-NHS-SS-biotin to enhance bacterial signal detection. This membrane-impermeable reagent covalently couples to primary amines (mainly lysine residues [46,47] and N-termini [48]) exposed on the bacterial surface, enabling selective enrichment of surface-associated proteins. However, this strategy may preferentially capture proteins with a higher density of accessible lysines, potentially leading to their overrepresentation, while proteins with fewer exposed amines or shielded within complexes may be underrepresented [30]. As a limitation of the study, it must be noticed that the proteoforms were not considered but the identifications were only based on canonical proteins or ORF products from genomic data to generate peptide mass finger printing. As indicated in Table S1 and Table 1, some proteins were identified based on one peptide only but following MS/MS fragmentation for validation. Despite these hurdles, our findings demonstrate the feasibility of in vivo proteomic analyses to capture bacterial adaptations during distinct infection stages. At the early stage of infection, EHEC exhibited significant upregulation of proteins associated with anaerobic metabolism and stress response, particularly in the colon. Enzymes such as glycerol-3-phosphate dehydrogenase (GlpB) and electron transport protein FixC were overrepresented, suggesting a rapid metabolic shift to anaerobic respiration in response to the hypoxic environment of the colon. The colon’s lower oxygen availability compared to the ileum imposes a strong selective pressure on EHEC, forcing the pathogen to utilise alternative electron acceptors for energy production. This metabolic shift is a well-described adaptation in other enteric pathogens, such as *Salmonella enterica* and *Shigella flexneri*, during intestinal colonisation [49,50]. Concurrently, molecular chaperones like GroEL and DnaK were identified, reflecting the physiological stress experienced by EHEC upon initial exposure to the host environment. These proteins play essential roles in protein folding and stabilisation, enabling the pathogen to survive the harsh conditions of the intestinal lumen, such as bile salts, osmotic stress, and antimicrobial peptides. The simultaneous upregulation of anaerobic metabolism and stress response proteins highlights EHEC’s rapid and coordinated adaptation to early-stage host pressures, ensuring survival and colonisation initiation.

By 10 h post-infection, the proteomic profile revealed a notable shift toward proteins involved in nutrient acquisition, outer membrane transport, and virulence factor expression. Outer membrane transporters, including OmpC and FecA, were identified, supporting the pathogen ability to scavenge iron and other essential nutrients under conditions of nutritional immunity. Iron acquisition is particularly critical during late infection stages, as the host actively restricts iron availability as a defense mechanism [51]. Upregulation of siderophore transport systems by EHEC mirrors adaptations observed in other pathogens like *Yersinia pestis* and *Vibrio cholerae*, which utilise similar mechanisms to counteract host iron sequestration [52,53]. Additionally, proteins involved in adhesion to host cells and virulence factor secretion, such as type III secretion system (T3SS) components, were downregulated at this stage, marking EHEC transition to intimate interactions with host epithelial cells. This suggests that by 10 h, EHEC has established a stable colonisation niche, prioritising nutrient uptake to ensure persistence. Such a temporal shift in proteomic abundance has been reported in *Salmonella*, where metabolic pathways and virulence factors are tightly regulated to balance energy conservation and host colonisation [49,54,55]. Together, these findings underscore EHEC capacity to fine-tune its surface proteome in response to both spatial (intestinal region) and temporal (infection stage) host-derived pressures. While cytoplasmic enzymes found at the bacterial cell surface can be informative of the bacterial physiological status, some of them can also moonlight at the bacterial cell surface and have a role in bacterial adhesion such as DnaK [56]. Consistent with this, several of the proteins annotated as cytoplasmic or metabolic in our dataset may represent moonlighting proteins that have been repeatedly reported at bacterial surfaces [8,57,58]. Nevertheless, we cannot fully exclude contributions from abundant cytoplasmic proteins co-purified during biotinylation, which is a known limitation of affinity-based enrichment strategies. These points should be considered when interpreting the dataset, but they do not diminish the biological relevance of the observed spatial- and temporal-specific changes.

Comparisons with previous studies highlight both conserved and pathogen-specific responses during enteric infection. For example, the upregulation of stress response proteins and anaerobic metabolic enzymes mirrors adaptations observed in other enteric pathogens, suggesting that these strategies are broadly conserved mechanisms for surviving in the gastrointestinal tract. In contrast, the pronounced regulation of iron acquisition systems and virulence-associated outer membrane proteins in EHEC reflects its specialised adaptations for nutrient acquisition and host colonisation. These findings highlight the importance of proteomic studies in uncovering not only universal bacterial survival strategies but also species-specific mechanisms that could be exploited for pathogen control. It should also be emphasized that comparable in vivo proteomic datasets remain extremely scarce, particularly those addressing bacterial surface proteomes during intestinal infection. As such, our dataset provides a valuable foundation for future cross-validation and benchmarking as more studies become available, thereby contributing to the collective effort of building a more comprehensive understanding of host–pathogen adaptation in vivo. Future studies integrating proteomic with lipidomic analyses will also be critical to better understand how membrane composition influences secretion pathways and surface adaptations, ultimately providing additional opportunities for therapeutic intervention.

This study provides a comprehensive characterisation of EHEC O157:H7 surface proteome dynamics during murine ileal loop infection, revealing the remarkable ability of the pathogen to adapt to spatial and temporal host environmental changes. Despite the challenges of in vivo proteomic analyses, our studies pave the way for further investigations of pathogen adaptation in the course of infection along the gastro-intestinal track and offer valuable insights for the identification of potential targets for therapeutic intervention and vaccine development.

## Figures and Tables

**Table 1:** Bacterial proteins with higher abundance identified in the colon and ileum at 3 h and 10 h post-infection. The table lists bacterial proteins that were detected with higher abundance during early (3 h) and late (10 h) infection in two regions of the gastrointestinal tract: colon and ileum. The Uniprot ID column provides the unique protein identifiers, while the Gene name column specifies the associated gene. The Function column describes the function (from Uniprot) of each protein, including roles in metabolism, energy production, protein synthesis, virulence, and stress adaptation. The columns Highest abundance conditions and Lowest abundance condition indicate the specific region (colon or ileum) and time point (3 h or 10 h) where the protein was more or less abundant, respectively.

Uniprot ID	Protein Name (Gene)	Function	Highest Abundance Conditions	Lowest Abundance Condition
Q8XE13	GlpB (*Z3500*)	Conversion of glycerol 3-phosphate to dihydroxyacetone. Uses fumarate or nitrate as electron acceptor.	Colon 3 h	Ileum 3 h
Q7AHT0	FixC (*Z0049*)	Could be part of an electron transfer system required for anaerobic carnitine reduction.	Colon 3 h	Ileum 3 h
P66607	RpsG (*Z4699*)	One of the primary rRNA binding proteins, it binds directly to 16S rRNA where it nucleates assembly of the head domain of the 30S subunit. Is located at the subunit interface close to the decoding center, probably blocks exit of the E-site tRNA.	Colon 3 h	Ileum 3 h
P0ABB6	AtpD (*Z5230*)	Produces ATP from ADP in the presence of a proton gradient across the membrane. The catalytic sites are hosted primarily by the beta subunits.	Colon 3 h	Ileum 3 h
A0A9Q6ZIS0	PuuC (*Z2488*)	Catalyses the aminotransferase reaction from putrescine to 2-oxoglutarate, leading to glutamate and 4-aminobutanal, which spontaneously cyclises to form 1-pyrroline.	Colon 3 h	Ileum 3 h
P0AD04	LetA (*Z2880*)	Lipophilic envelope-spanning tunnel protein AComponent of a transport pathway that contributes to membrane integrity.	Colon 3 h	Ileum 3 h
P62401	RplE (*Z4678*)	This is one of the proteins that bind and probably mediate the attachment of the 5S RNA into the large ribosomal subunit, where it forms part of the central protuberance. In the 70S ribosome it contacts protein S13 of the 30S subunit (bridge B1b), connecting the 2 subunits; this bridge is implicated in subunit movement. Contacts the P site tRNA; the 5S rRNA and some of its associated proteins might help stabilise positioning of ribosome-bound tRNAs.	Colon 3 h	Colon 10 h
P0A7W0	RpsD (*Z4666*)	Functions as a rho-dependent antiterminator of rRNA transcription, increasing the synthesis of rRNA under conditions of excess protein, allowing a more rapid return to homeostasis. Binds directly to RNA polymerase	Colon 3 h	Ileum 3 h
A0A5Q2ENA4	CydC (*Z1230*)	Maintain the reduced state of cytoplasmic L-cysteine, thereby providing an important connection between sulfur metabolism, oxidative stress and resistance to antibiotics	Colon 3 h	Ileum 3 h
A0A9Q7EDA9	GdhA (*Z2793*)	Catalyses the reversible oxidative deamination of glutamate to alpha-ketoglutarate and ammonia.	Colon 3 h	Ileum 10 h
P0A8T8	RpoC (*Z5561*)	DNA-dependent RNA polymerase catalyses the transcription of DNA into RNA using the four ribonucleoside triphosphates as substrates.	Colon 3 h	Ileum 10 h
P0A7E7	PyrG (*Z4095*)	Catalyses the ATP-dependent amination of UTP to CTP with either L-glutamine or ammonia as the source of nitrogen. Regulates intracellular CTP levels through interactions with the four ribonucleotide triphosphates.	Ileum 3 h	Ileum 10 h
Q8XEC3	UbiC (*Z5638*)	Removes the pyruvyl group from chorismate, with concomitant aromatisation of the ring, to provide 4-hydroxybenzoate (4HB) for the ubiquinone pathway.	Ileum 3 h	Colon 10 h
Q8XDH7	TreA (*Z1968*)	Provides the cells with the ability to utilise trehalose at high osmolarity by splitting it into glucose molecules that can subsequently be taken up by the phosphotransferase-mediated uptake system.	Ileum 3 h	Ileum 10 h
A0A9Q7EEZ3	EivE (*Z4196*)	Encoded within the type III secretion system (SPI-1 T3SS), it is essential for the translocation of protein effectors into host cells. Forms a complex with SipB and SipC in the presence of their chaperone SicA. Positively regulates the secretion of SPI-1 T3SS effector proteins SipB, SipC and SipD and negatively influences the secretion of SipA, SopA and SptP.	Ileum 3 h Colon 3 h	Colon 10 h
A0A9Q6Z841	MreC (*Z4609*)	Involved in formation and maintenance of cell shape. Responsible for formation of rod shape. May also contribute to regulation of formation of penicillin-binding proteins.	Ileum 3 h Colon 3 h	Colon 10 h
Q8X8S5	Csm (*Z2389*)	DNA (cytosine-5-)-methyltransferase activity, acting on CpNpG substrates.	Ileum 3 h Colon 3 h	Colon 10 h
Q8XBL5	LigA (*Z3677*)	DNA ligase that catalyses the formation of phosphodiester linkages between 5′-phosphoryl and 3′-hydroxyl groups in double-stranded DNA using NAD as a coenzyme and as the energy source for the reaction. It is essential for DNA replication and repair of damaged DNA.	Ileum 10 h	Ileum 3 h
P0AC43	SdhA (*Z0877*)	Two distinct, membrane-bound, FAD-containing enzymes are responsible for the catalysis of fumarate and succinate interconversion; the fumarate reductase is used in anaerobic growth, and the succinate dehydrogenase is used in aerobic growth.	Ileum 10 h	Ileum 3 h
P0ABB2	AtpA (*Z5232*)	Produces ATP from ADP in the presence of a proton gradient across the membrane. The alpha chain is a regulatory subunit.	Ileum 10 h	Ileum 3 h
Q8X8G9	YihI (*Z5402*)	A GTPase-activating protein (GAP) that modifies Der/EngA GTPase function. May play a role in ribosome biogenesis.	Ileum 10 h	Ileum 3 h
P64258	MraY (*Z0097*)	Catalyses the initial step of the lipid cycle reactions in the biosynthesis of the cell wall peptidoglycan: transfers peptidoglycan precursor phospho-MurNAc-pentapeptide from UDP-MurNAc-pentapeptide onto the lipid carrier undecaprenyl phosphate, yielding undecaprenyl-pyrophosphoryl-MurNAc-pentapeptide, known as lipid I.	Ileum 10 h	Ileum 3 h
P0AA94	YpdA (*Z3645*)	Member of the two-component regulatory system YpdA/YpdB. YpdA activates YpdB by phosphorylation in response to high concentrations of extracellular pyruvate.	Ileum 10 h	Ileum 3 h
Q8X643	PreA (*Z3402*)	Involved in pyrimidine base degradation. Catalyses physiologically the reduction in uracil to 5,6-dihydrouracil (DHU) by using NADH as a specific cosubstrate. It also catalyses the reverse reaction and the reduction in thymine to 5,6-dihydrothymine (DHT).	Ileum 10 h	Ileum 3 h
Q8X6J9	LysRA (*Z0346*)	Regulates the expression of the nodABCFE genes which encode other nodulation proteins.	Ileum 10 h Colon 10 h	Colon 3 h
P0A869	TalA (*Z3720*)	Transaldolase is important for the balance of metabolites in the pentose-phosphate pathway.	Ileum 10 h Colon 10 h	Ileum 3 h
A0A9Q7ED38	RcsD (*Z3475*)	Component of the Rcs signaling system, which controls transcription of numerous genes. RcsD is a phosphotransfer intermediate between the sensor kinase RcsC and the response regulator RcsB. It acquires a phosphoryl group from RcsC and transfers it to RcsB.	Ileum 10 h Colon 10 h	Colon 3 h
Q8X654	LysRB (*Z3395*)	Regulatory gene, a lysR homologue, which regulates the level of *chvE*	Ileum 10 h Colon 10 h	Ileum 3 h

## Data Availability

The mass spectrometry proteomics data underlying this study have been deposited to the MassIVE repository with the dataset identifier MSV000098641. The dataset includes peak list files in mzML format and protein identification tables in Excel format. All files can be accessed at https://massive.ucsd.edu (accessed on 24 July 2025) under the dataset title “Surface proteome profiling of *E. coli* O157:H7 during in vivo murine infection (ID0243)”.

## Supplementary Materials

The following supporting information can be downloaded at https://www.mdpi.com/article/10.3390/proteomes13040052/s1, Table S1: Total of unique proteins of *E. coli* O157:H7 EDL933 identified in the 4 experimental conditions (Ileum 3 h and 10 h and Colon 3 h and 10 h).
